# Impact of in situ simulation training on quality of postnatal stabilization and resuscitation—a before-and-after, non-controlled quality improvement study

**DOI:** 10.1007/s00431-024-05781-3

**Published:** 2024-09-23

**Authors:** Lukas P. Mileder, Nariae Baik-Schneditz, Jasmin Pansy, Bernhard Schwaberger, Wolfgang Raith, Alexander Avian, Georg M. Schmölzer, Peter Wöckinger, Gerhard Pichler, Berndt Urlesberger

**Affiliations:** 1 Division of Neonatology, Department of Paediatrics and Adolescent Medicine, Medical University of Graz, Auenbruggerplatz 34/2, 8036 Graz, Austria; 2 Paediatric Simulation Group Graz, Department of Paediatrics and Adolescent Medicine, Medical University of Graz, Graz, Austria; 3 Institute for Medical Informatics, Statistics and Documentation, Medical University of Graz, Graz, Austria; 4 Northern Alberta Neonatal Program, University of Alberta, Edmonton, Canada; 5 Centre for the Studies of Asphyxia and Resuscitation, Royal Alexandra Hospital, Edmonton, Canada; 6 IIDepartment of Paediatrics II, Neonatology, Medical University of Innsbruck, Innsbruck, Austria

**Keywords:** Neonate, Preterm, Postnatal stabilization, Resuscitation, Video recording, Non-technical skills, Simulation training, In situ training

## Abstract

This study aimed to evaluate the impact of in situ simulation-based training on quality indicators of patient care at a level IV neonatal intensive care unit. A before-and-after, non-controlled quality improvement study was performed at the Division of Neonatology, Medical University of Graz. The educational intervention comprised a period of 4 months, with structured in situ simulation training delivered regularly for neonatal providers and nurses in interprofessional teams. The primary study outcome was the quality of non-technical skills and team interaction during actual postnatal stabilization and resuscitation. This was assessed using video recording during two 2-month observational phases before (pre-training) and after the educational intervention (post-training). Delivery room video recordings were assessed by two external, blinded neonatologists using the Anaesthetists’ Non-Technical Skills (ANTS) score. Furthermore, we collected clinical patient data from video-recorded neonates during the pre- and post-training periods, and training participants’ individual knowledge of neonatal resuscitation guidelines was assessed using a before- and after-questionnaire. A total of 48 healthcare professionals participated in 41 in situ simulation trainings. The level of non-technical skills and team interaction was already high in the pre-training period, and it did not further improve afterwards. Nonetheless, we observed a significant increase in the teamwork event “evaluation of plans” (0.5 [IQR 0.0–1.0] versus 1.0 [1.0–2.0], *p* = 0.049). Following the educational intervention, training participants’ knowledge of neonatal resuscitation guidelines significantly improved, although there were no differences in secondary clinical outcome parameters.

*Conclusion*: We have successfully implemented a neonatal in situ simulation training programme. The observed improvement in one teamwork event category in the post-training period demonstrates the effectiveness of the training curriculum, while also showing the potential of in situ simulation training for improving postnatal care and, ultimately, patient outcome.

**Table:** 

**What is Known:** • * In situ simulation-based training is conducted in the real healthcare environment, thus promoting experiential learning which is closely aligned with providers’ actual work.* • * In situ simulation-based training may offer an additional benefit for patient outcomes in comparison to other instructional methodologies.*
**What is New:** • *This observational study investigated translational patient outcomes in preterm neonates before and after delivery of high-frequency in situ simulation-based training at a level IV neonatal intensive care unit.* • *There was a significant increase in the frequency of one major teamwork event following the delivery of in situ simulation-based training, indicating a notable improvement in the non-technical skills domain, which is closely linked to actual team performance.*

**What is Known:**

• * In situ simulation-based training is conducted in the real healthcare environment, thus promoting experiential learning which is closely aligned with providers’ actual work.*

• * In situ simulation-based training may offer an additional benefit for patient outcomes in comparison to other instructional methodologies.*

**What is New:**

• *This observational study investigated translational patient outcomes in preterm neonates before and after delivery of high-frequency in situ simulation-based training at a level IV neonatal intensive care unit.*

• *There was a significant increase in the frequency of one major teamwork event following the delivery of in situ simulation-based training, indicating a notable improvement in the non-technical skills domain, which is closely linked to actual team performance.*

## Introduction

Simulation-based training (SBT) is ideally suited for “*dynamic domains involving high hazard and invasive intervention*”, including, but not limited to, anaesthesia, critical care, and emergency medicine [1]. Due to the challenges and risks associated with the care for depressed or non-vigorous neonates, particularly immediately after birth, neonatal-perinatal medicine is recognized as a discipline with substantial potential for SBT. SBT offers a safe “arena” for structured learning tailored to participants’ needs, facilitating active experimentation which is fundamental for adult learning. It also integrates the practice of cognitive, technical, and behavioural, non-technical skills, which are essential for interdisciplinary and interprofessional training [2–4]. Recognized as a “*promising tool to improve patient safety, team performance, and ultimately patient outcomes*” [5], simulation-based educational techniques have developed into an integral element of neonatal-perinatal training in many institutions [6, 7]. Hence, current neonatal resuscitation guidelines explicitly recommend SBT as well as team and leadership training as part of educational programmes [8].

SBT delivered in the real healthcare environment, with on-duty providers as training participants, has been coined in situ simulation [9]. It offers advantages over centre-based training, by promoting experiential learning closely aligned with healthcare providers’ actual work and improving training efficiency for institutions and participants [9]. In situ SBT can also be used to analyze and improve the quality of patient care, to identify latent safety threats in actual healthcare environments, and to test new facilities [10–12]. In addition to these benefits, a recent systematic review and meta-analysis reported an improvement in patient mortality and morbidity when comparing in situ SBT with current educational practices [13]. However, so far only a few studies have investigated the stringent use of in situ SBT and its impact on the quality of postnatal stabilization and resuscitation, which was the aim of the present study.

## Materials and methods

We performed a before-and-after, non-controlled quality improvement study at the Division of Neonatology, Department of Paediatrics and Adolescent Medicine, Medical University of Graz, Austria, a level IV neonatal intensive care unit [14] covering approximately 3500 inborn births per year. The study was approved by our university’s Ethics Committee (27–014 ex 14/15). Physicians (residents, fellows, and consultants) and nurses at the Division of Neonatology were invited to participate on a voluntary basis and informed consent was obtained prior to study participation.

### In situ SBT

Unannounced in situ SBT was delivered over a period of 4 months, aiming at three to five training sessions every week. Trainings were delivered based on clinical workload to ensure dedicated, uninterrupted participation. A high-fidelity neonatal simulator (Gaumard Newborn HAL S3010, Gaumard Scientific Company Inc., Miami, United States of America) was used for SBT in the resuscitation room and clinical area of the Division of Neonatology. The training sessions were delivered by one simulation trainer (LPM) and scenarios focused on key challenges and typical clinical situations encountered during postnatal care. Each training session comprised 30 to 50 minutes, with 15–20 minutes of interprofessional SBT followed by 15–30 minutes of structured debriefing [15].

### Outcome measures

The primary outcome was the quality of non-technical skills and team interaction during actual postnatal stabilization and resuscitation. This was assessed using the Anaesthetists’ Non-Technical Skills (ANTS) score, which comprises four main categories (Task Management, Team Working, Situation Awareness, Decision Making) and a total of 15 individual elements, with scores ranging from 1 to 4 (1 = poor, 2 = marginal, 3 = acceptable, 4 = good, n = not observed) [16]. A recent systematic review found a substantial interrater reliability in the majority of studies that employed the ANTS score [17]. As a further outcome measure, the number of five specific teamwork events (sharing information, inquiry, assertion, teaching/advising, and evaluation of plans) was also determined from video review [18].

Assessments took place during two 2-month observational phases before (pre-training) and after delivery of in situ SBT (post-training). During these phases, postnatal stabilization and resuscitation of preterm and term neonates, without major congenital cardio-pulmonary malformations who required respiratory and/or cardio-circulatory support after birth, was video-recorded after parental consent. For video recordings, one installed camera targeted the neonate on the resuscitation table or in the incubator, while a second mobile camera recorded the healthcare team during the provision of postnatal care. To analyze the primary outcome, two external neonatologists (GMS, PW) reviewed all the recordings independently and in random order using the ANTS score, focussing on team performance rather than on individual performance.

Furthermore, demographic and clinical data of the video-recorded neonates were collected as secondary outcome measures either from video recordings, our unit’s polygraphic data management system (Alpha-Trace digital MM, B.E.S.T. Medical Systems, Vienna, Austria) or patient charts. Among those were as follows:

- time from neonate’s arrival at the resuscitation table to heart rate (HR) auscultation (seconds)
- time from neonate’s arrival at the resuscitation table to first ventilation breath (if required) (seconds)
- number of endotracheal intubation attempts (if required) during postnatal stabilization and resuscitation
- arterial oxygen saturation (SpO_2_) and HR 5 min after the neonate’s arrival at the resuscitation table (% and beats per minute, respectively)
- rectal body temperature during/immediately after postnatal stabilization and resuscitation (°C)
- Apgar scores at minutes 1, 5, and 10 after birth
- development of pneumothorax requiring chest tube insertion within 24 h after birth
- length of hospitalization (days)
- in-hospital mortality

Finally, training participants’ individual knowledge of neonatal resuscitation guidelines was assessed before and after in situ SBT. For this purpose, participants individually completed a standardized, 20-question paper-and-pencil knowledge test on neonatal resuscitation guidelines, developed by the European Resuscitation Council for its Newborn Life Support provider courses.

### Sample size calculation and statistical analyses

Sample size calculation was based on the study by Thomas et al. [18], in which adding additional teamwork training to standard Neonatal Resuscitation Program training resulted in a mean of 3.8 more teamwork events (i.e. sharing information, inquiry, assertion, teaching/advising, evaluation of plans) in the intervention group. According to our previous experiences, we assumed that approximately 30 neonates could be recorded during the pre- and post-training periods. Assuming comparable differences between the pre- and post-training periods as mentioned above and using the same standard deviation (SD: 3.4), a similar effect could be detected in a study with 30 pre- and post-training period events of postnatal stabilization and resuscitation, when using a *t*-test for independent samples with a power of 98%.

Data are presented as frequencies, absolute and relative values, mean ± SD, or median (IQR), as appropriate. Shapiro–Wilk and Kolmogorov–Smirnov tests were used to test for normal distribution. Since it was not possible to record the names of all team members of each event of postnatal stabilization and resuscitation, tests for independent data were used to compare pre- and post-training periods. Sex, administration of prenatal steroids, clinical signs of chorioamnionitis, the need for respiratory support after birth, frequency of hypothermia, and development of pneumothorax were compared between the pre- and post-training periods using either Chi-square test or Fisher’s exact test. Results of the pre- and post-cognitive knowledge tests were compared using the Wilcoxon signed-rank test. Depending on data distribution, the *t*-test for independent samples or the Mann–Whitney *U* test was used to compare the remaining outcome measures, including the primary study outcome. A *p*-value of < 0.05 was considered statistically significant. Statistical analyses were performed using IBM SPSS Statistics 28 (Armonk, United States of America).

## Results

Forty-one in situ simulation trainings were delivered over the study period of 4 months, corresponding to a mean of 2.6 trainings per week, excluding the 2 weeks of the Christmas holidays. Twenty-one physicians and 27 nurses participated in the study, with a median of two physicians (1–3) and two neonatal nurses (2–3) in each training session. Simulation scenarios covered peri- and postnatal asphyxia (*n* = 11), meconium aspiration syndrome (*n* = 9), respiratory distress and postnatal management of preterm neonates (*n* = 8), early-onset bacterial infection or sepsis (*n* = 4), congenital cardiac defect (*n* = 3), neonatal seizures (*n* = 3), difficult airway (*n* = 2), and pneumothorax (*n* = 1).

### Patients (pre- and post-training periods)

Twenty preterm neonates participated in a clinical study at the Division of Neonatology Graz during the pre-training period. Of those, 15 neonates (75.0%) required medical support after birth. During the post-training period, 13 of 25 video-recorded neonates (52.0%) were in need of medical support after birth and, thus, were included in this study.

Demographic data of these 28 neonates, who were all delivered by Caesarean section, are presented in Table 1. Birth weight was lower in the post-training period, but gestational age and sex did not differ between the groups.

**Table 1:** Demographic data of the included 28 neonates (absolute [relative] numbers or mean ± SD, as appropriate) (* = *p* < 0.05)

	All neonates (*n* = 28)	Pre-training (*n* = 15)	Post-training (*n* = 13)	*p*-value
Gestational age (weeks)	30.4 ± 3.6	31.5 ± 3.7	29.2 ± 3.3	0.101
Male sex (%)	16 (57.1)	9 (60)	7 (53.8)	0.743
Birth weight (grams)	1441 ± 672	1678 ± 744	1168 ± 469	0.043*
Prenatal steroids (%)	23 (82.1)	13 (86.7)	10 (76.9)	0.502
Clinical signs of chorioamnionitis (%)	3 (10.7)	2 (13.3)	1 (7.7)	0.630
Caesarean section (%)	28 (100)	15 (100%)	13 (100%)	-

All 28 neonates required respiratory support after birth. There were no differences in either oropharyngeal suctioning (80.0% versus 76.9%, *p* = 1.000), oxygen supplementation (93.3% versus 84.6%, *p* = 0.583), mask continuous positive airway pressure (100% versus 100%), mask positive pressure ventilation (60.0% versus 76.9%, *p* = 0.435), or the need for intubation (26.7% versus 53.8%, *p* = 0.142) between the pre- and post-training period.

### Primary study outcome

Video recordings were available from 12 of 15 neonates (80.0%) and from 13 of 13 neonates (100%) during the pre- and post-training periods, respectively. The median duration of video recordings did not differ between the pre- and post-training periods (15 [10.25–15.0] versus 15 [15.0–15.0] min, *p* = 0.393).

**Fig. 1:**
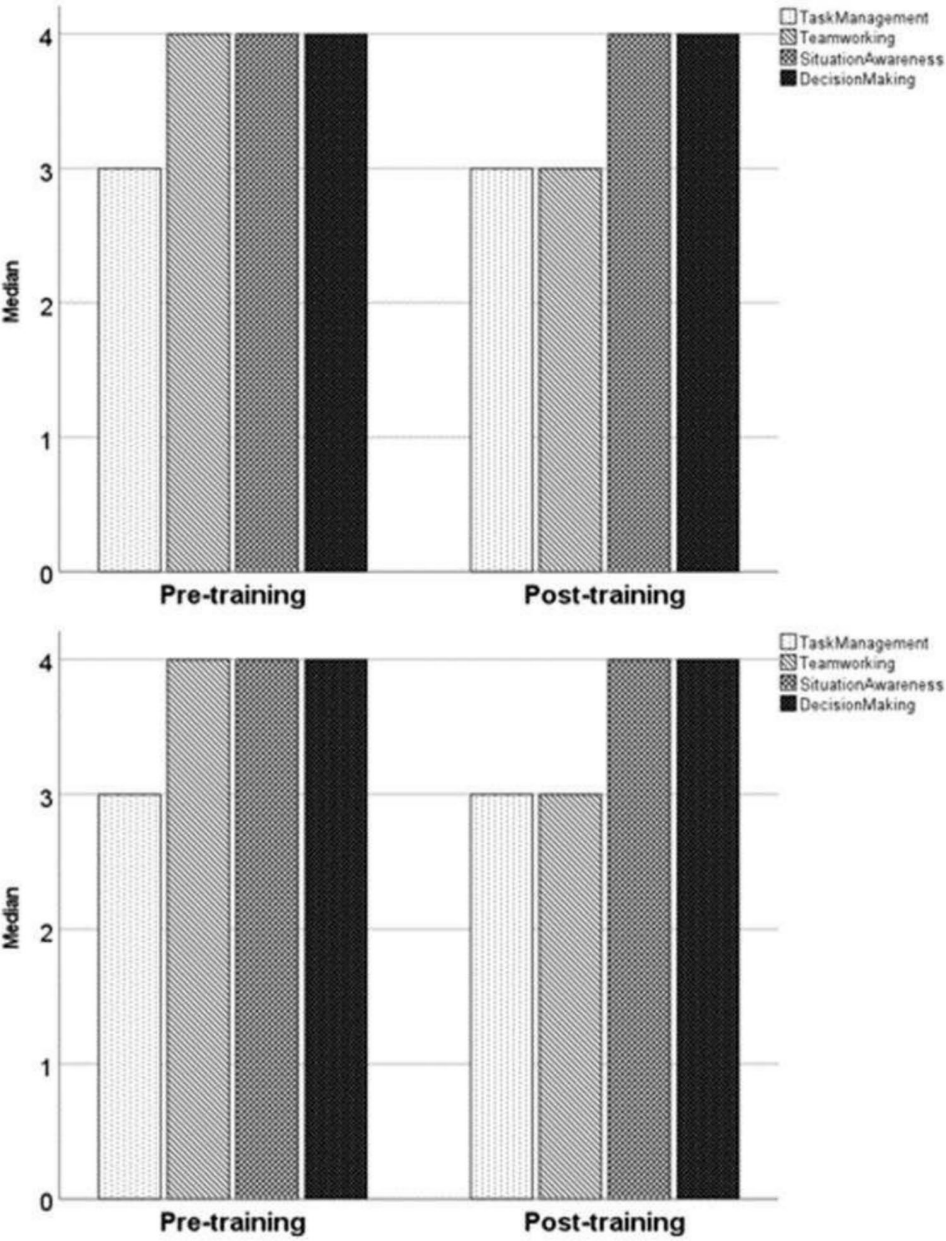
Anaesthetists’ Non-Technical Skills (ANTS) categories for the pre- (left columns) and post-training periods (right columns), according to the first (upper figure) and second (lower figure) video evaluator (4 = good, 3 = acceptable, 2 = marginal, 1 = poor)

According to both video evaluators, there were no differences in either the four main ANTS categories (Fig. 1) or in any of the 15 ANTS elements (Table 2). In general, the level of non-technical skills and team interaction was already high in the pre-training period, with three of the four main ANTS categories having a “good” evaluation before implementation of in situ SBT, as rated by both evaluators (Fig. 1).

**Table 2:** Individual Anaesthetists’ Non-Technical Skills (ANTS) elements (median scores [IQR]) for pre- and post-training periods, according to first (left) and second video evaluator (4 = good, 3 = acceptable, 2 = marginal, 1 = poor)

ANTS elements	First video evaluator			Second video evaluator		
	Pre-training	Post-training	*p*-value	Pre-training	Post-training	*p*-value
Planning and preparing	2.5 (2.0–3.0)	(No scoring)	-	4.0 (2.25–4.0)	3.0 (2.0–4.0)	0.695
Prioritizing	3.0 (2.75–4.0)	4.0 (2.0–4.0)	*p* = 0.832	4.0 (3.0–4.0)	4.0 (2.0–4.0)	0.400
Providing and maintaining standards	4.0 (4.0–4.0)	4.0 (3.0–4.0)	*p* = 0.207	4.0 (3.0–4.0)	4.0 (2.0–4.0)	0.743
Identifying and utilizing resources	3.0 (2.0–3.0)	4.0 (2.0–4.0)	*p* = 0.392	4.0 (3.5–4.0)	4.0 (2.0–4.0)	0.731
Co-ordinating activities with team members	3.0 (2.0–4.0)	4.0 (2.0–4.0)	*p* = 0.494	4.0 (3.0–4.0)	4.0 (2.5–4.0)	0.303
Exchanging information	2.0 (2.0–3.0)	4.0 (2.0–4.0)	*p* = 0.093	4.0 (3.0–4.0)	4.0 (3.0–4.0)	0.605
Using authority and assertiveness	3.0 (1.0–4.0)	3.0 (2.5–4.0)	*p* = 0.649	4.0 (3.0–4.0)	4.0 (3.0–4.0)	1.000
Assessing capabilities	3.0 (2.0–4.0)	3.0 (2.5–4.0)	*p* = 0.531	4.0 (4.0–4.0)	4.0 (3.5–4.0)	0.481
Supporting others	3.0 (1.0–4.0)	3.0 (1.0–4.0)	*p* = 0.865	4.0 (3.0–4.0)	4.0 (4.0–4.0)	0.691
Gathering information	3.0 (1.0–3.0)	3.0 (2.0–4.0)	*p* = 0.531	4.0 (3.5–4.0)	4.0 (4.0–4.0)	0.602
Recognizing and understanding	3.0 (2.0–4.0)	3.0 (2.0–4.0)	*p* = 0.820	4.0 (3.0–4.0)	4.0 (3.25–4.0)	0.422
Anticipating	2.5 (1.75–4.0)	2.0 (2.0–4.0)	*p* = 0.738	4.0 (3.25–4.0)	4.0 (3.75–4.0)	0.897
Identifying options	3.0 (1.75–3.0)	3.0 (2.0–4.0)	*p* = 0.376	4.0 (4.0–4.0)	4.0 (3.5–4.0)	0.529
Balancing risks and selecting options	2.0 (2.0–3.0)	3.0 (2.0–4.0)	*p* = 0.303	4.0 (3.25–4.0)	4.0 (4.0–4.0)	0.686
Re-evaluating	3.0 (2.0–4.0)	3.0 (3.0–4.0)	*p* = 0.733	4.0 (4.0–4.0)	4.0 (3.25–4.0)	0.524

**Fig. 2:**
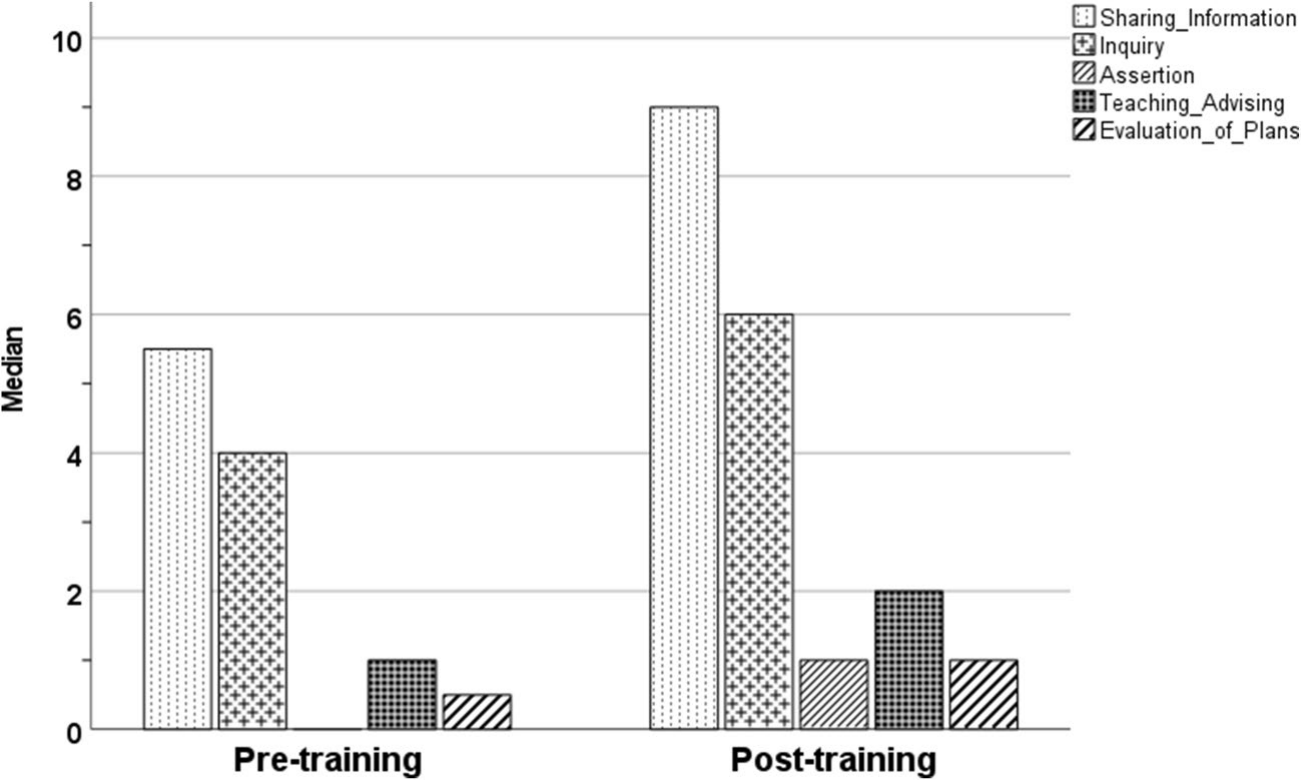
Teamwork events per neonate (median) during the pre- and post-training periods (sharing information, *p* = 0.102; inquiry, *p* = 0.088; assertion, *p* = 0.129; teaching/advising, *p* = 0.911; evaluation of plans, *p* = 0.049)

There was a significant increase in the frequency of the teamwork event “evaluation of plans” (0.5 [0.0–1.0] versus 1.0 [1.0–2.0], *p* = 0.049) during the post-training period, while the incidence of the other four teamwork events did not increase significantly (Fig. 2). The total number of all five teamwork events increased from 15.0 (10.0–24.3) to 18.0 (13.5–30.5) after the delivery of in situ SBT, but without reaching statistical significance (*p* = 0.056; Fig. 2).

### Secondary study outcomes

When comparing the clinical outcome parameters, there were no differences between the pre- and post-training period, except for significantly lower 5-minute Apgar scores (9 [8–9] versus 8 [8–9], *p* = 0.045). However, after removing the three deceased neonates (one in the pre-training period and two in the post-training period) from the analysis, 5-minute Apgar scores did not differ significantly (9 [8–9] versus 8 [8–9], *p* = 0.191). Secondary outcome parameters are detailed in Table 3.

**Table 3:** Secondary study outcomes for the pre- and post-training periods (absolute values, mean ± SD or median (IQR), as appropriate) (* = *p* < 0.05)

	Pre-training	Post-training	*p*-value
Time from arrival at the resuscitation table to HR auscultation (seconds)	9.5 (3.0–22.75)	10.0 (5.25–21.75)	0.715
Time from arrival at the resuscitation table to first ventilation breath (seconds)	27.0 (16.5–43.0)	30.0 (14.0–49.0)	0.871
Number of endotracheal intubation attempts (*n*)	2 (1.25–2.75)	2 (1–3)	0.920
SpO_2_ 5 minutes after arrival at the resuscitation table (%)	78.5 (72.25–86.25)	76 (62–84)	0.406
HR 5 minutes after arrival at the resuscitation table (beats per minute)	137.5 (128.5–147.0)	144 (93–152)	0.732
Rectal body temperature (°C)	36.8 ± 0.2	36.6 ± 0.7	0.394
Apgar at 1 minute	7 (6–8)	6 (4.5–8)	0.421
Apgar at 5 minutes	9 (8–9)	8 (7–9)	0.045*
Apgar at 10 minutes	9 (9–9)	9 (9–9)	0.697
Pneumothorax within 24 h after birth (*n*)	1/15	0/13	1.000
Length of hospitalization (days)	28 (16–57)	41 (15–75.5)	0.447
Mortality (*n*)	1/15	2/13	0.583

*HR* heart rate, *SpO*_*2*_ arterial oxygen saturation

Twenty-nine of the 48 training participants (60.4%) answered the standardized cognitive knowledge test before and after in situ SBT, showing a significant increase in correctly answered questions between the pre- and post-training periods (15 [13–15.5] versus 18 [17–19], *p* < 0.001).

## Discussion

This study is among the first investigating the impact of a dedicated in situ simulation training programme on neonatal short-term outcome, including the length of hospitalization and in-hospital mortality. In the context of translational science [19], this study assessed T2 outcomes (“*evidence of clinical effectiveness at the level of the patient”*) and T3 outcomes (“*health care delivery …and preventive services that yield measureable improvements in the health of individuals and society”*) [19], which is important to inform best educational practices.

During the intervention period, we aimed at delivering three to five training sessions every week. Although we did not achieve this ambitious goal, we managed to deliver an average of 2.6 trainings per week over the 4-month period. Patient care responsibilities and clinical obligations, which have also been reported by other in situ SBT programmes [20, 21], were observed as the main obstacles for training delivery and staff participation.

Neither of the two video evaluators identified differences in the four ANTS categories (Task Management, Team Working, Situation Awareness, and Decision Making) or in any of their 15 individual elements. However, when interpreting the results, one must bear in mind the already high level of non-technical skills and team interaction observed during the pre-training period, which offered only little room for further improvement. This high level before the delivery of our educational intervention is even more remarkable, as there was no dedicated team training curriculum in place at our institution before this project. Still, there was a significant improvement in the teamwork event “evaluation of plans” during the post-training period. Although a causal link cannot be established, this improvement could be attributed to the delivered in situ SBT, which mainly targeted interprofessional and interdisciplinary teamwork and communication. Regular re-evaluation of the clinical situation and the diagnostic and therapeutic plan is essential to avoid fixation errors and is recognized as a key element of situational awareness, a common attribute of high-performing healthcare teams [22, 23]. Our finding of an improvement in the teamwork domain could actually result in improved patient care practices. Studies across different disciplines have shown that teamwork training is associated with improved protocol adherence and reduced activation times of extracorporeal cardiopulmonary resuscitation teams [24], shortened door-to-needle time in stroke patients requiring thrombolysis [25], and fewer maternal blood transfusions and postpartum bleeding events [26]. Accordingly, Brogaard et al. [27] reported a 5.5-fold increase of the likelihood of high clinical performance during postnatal stabilization and resuscitation in case of excellent non-technical skills execution.

Following the delivery of in situ SBT, the combined number of all five targeted teamwork events was increased, which almost reached statistical significance in our study. Higher teamwork event rates have been shown to be associated with faster, more efficient performance of simulated neonatal resuscitation [18], again emphasizing the potential relevance of this finding for postnatal care.

We did not find any differences in clinical secondary outcome parameters when comparing the pre- and post-training periods. Both time to HR assessment by auscultation and time to first ventilation breath were within the recommended time frames [8]. SpO_2_ and HR, assessed 5 minutes after arrival at the resuscitation table, did not show any differences between the pre- and post-training periods. Yet, during both observation periods, the median SpO_2_ was below the recommended value of 85% [8], which could be associated with higher odds of intraventricular haemorrhage [28]. While this finding illustrates the general challenge of SpO_2_ targeting, SpO_2_ values at 10 minutes were within recommended ranges during both observational phases (pre-training: 89.5% [83.00–91.25]; post-training: 90% [81–94]).

A total of 93.3% and 61.5% of preterm neonates in the pre- and post-training periods, respectively, maintained a normal body temperature (i.e. 36.5–37.5°C) during or immediately after postnatal stabilization and resuscitation. Although the insignificantly higher incidence of mild to moderate hypothermia in the post-training group requires attention, this finding is likely attributable to the significantly lower birth weight, which is inversely correlated with the frequency of postnatal hypothermia [29].

The finding of significantly lower 5-minute Apgar scores during the post-training period must be interpreted with caution. First, 1- and 10-minute Apgar scores did not differ between the two observational phases. Second, there is a positive correlation between birth weight and the 5-minute Apgar score in low- and very-low-birth-weight infants [30]. Third, the Apgar score exhibits poor interobserver reliability, especially in preterm neonates [31, 32]. And finally, after correcting for the three deceased neonates, no significant difference in 5-minute Apgar scores was observed.

Despite the lower birth weights of preterm neonates in the post-training period, there were no differences in the incidence of pneumothoraces, length of hospitalization, and in-hospital mortality. Even after adjusting the statistical analysis for the three extremely preterm neonates who had died within the first 9 days after birth, the duration of hospitalization did not differ between the two observational phases. In contrast, Theilen and colleagues [33] found an association between in situ SBT and fewer admissions to and shorter stays in a paediatric intensive care unit. Regarding patient survival, paediatric studies were able to show an association between simulation-based mock code training and a significant increase in survival after cardiopulmonary arrest [34] as well as between SBT combined with a debriefing of actual cardiac arrest events and improved in-hospital cardiopulmonary resuscitation [35].

Training participants’ knowledge of neonatal resuscitation guidelines significantly improved after the delivery of in situ SBT. Many studies have reported enhancements in the cognitive domain following simulation-based education [36, 37]. However, when comparing SBT to non-simulation-based educational interventions, there seems to be no additional benefit regarding knowledge acquisition [38], emphasizing the primary focus of SBT, i.e. acquisition and practice of technical and non-technical skills [2].

### Limitations

We must acknowledge several limitations of this study. First, we were unable to achieve the target of 30 events of postnatal stabilization and resuscitation during each period, as determined by the sample size calculation. The main reasons for this were parental refusal to participate and a below-average number of neonates requiring postnatal stabilization and resuscitation during the study period. Second, we performed an uncontrolled before-after study and were, therefore, not able to rule out any selection bias [39]. A controlled before-after study design, an interrupted time series, or a repeated measurement study could have helped mitigate this potential bias [39]. However, we performed our educational intervention over a rather short period of time and aimed at including all neonates requiring postnatal stabilization and resuscitation during the pre- and post-training periods, thus reducing the potential of selection bias. Third, video-recording may have influenced participants’ actual clinical behaviour [40]. Fourth, the video evaluators could not sufficiently assess the ANTS element “planning and preparing” in every video, because in some high-acuity deliveries team briefings were not fully video-recorded. Fifth, the ANTS score has not been developed or validated specifically for postnatal stabilization and resuscitation, in contrast to other more recently developed teamwork assessment tools such as the Global Assessment of Team Performance checklist [27]. Finally, we cannot rule out that the significantly lower birth weight of the preterm neonates in the post-training period interfered with our finding of a higher incidence of the teamwork event “evaluation of plans”, as these more vulnerable patients may have required a higher number of medical interventions.

## Conclusions

We have successfully conceptualized, implemented, and delivered an in situ simulation training programme with almost three training sessions per week over a 4-month period at our institution. Although we did not find an improvement in the quality of non-technical skills and team interaction following the delivery of in situ SBT, we observed an already high level during the pre-training period. Still, the frequency of the teamwork event “evaluation of plans” was significantly higher after our educational intervention, as was training participants’ knowledge of neonatal resuscitation guidelines. While these improvements could have a positive impact on the quality and effectiveness of postnatal care, controlled studies are still needed to strengthen the scientific foundation supporting simulation-based neonatal team trainings.

## Data Availability

The data that support the findings of this study are not openly available due to reasons of sensitivity and are available from the corresponding author upon reasonable request.

## Abbreviations

ANTS: Anaesthetists’ Non-Technical Skills
IQR: Interquartile range
HR: Heart rate
SD: Standard deviation
SBT: Simulation-based training
SpO_2_: Arterial oxygen saturation
